# Concomitant laparoscopic procedures can be done for urological diseases

**DOI:** 10.4103/0972-9941.65167

**Published:** 2010

**Authors:** Viroj Wiwanitkit

**Affiliations:** Wiwanitkit House, Bangkhae, Bangkok, Thailand 10160

Dear Sir,

With great interest, I read the recent publication on 'using concomitant laparoscopic procedures for urological diseases' by Maurya *et al*.[1] Maurya *et al*. have concluded that "Simultaneous laparoscopic procedures can be done for urological diseases in selected patients, with the advantages of single anaesthesia and hospital admission without increasing the morbidity".[1] Indeed, the concomitant laparoscopic procedure is accepted as a useful minimal access surgical procedure that can decrease the number of procedures from two major surgeries to only one minimal access surgery. In the study, there are some weak points. These include a) few numbers of subjects, especially when allocated in groups; b) lack of matched controls (same age and sex); and c) limitation due to the retrospective nature of the study. However, the results obtained from this study are useful and support the concept of using two minimal access procedures instead of two major surgeries. An interesting question is whether the concomitant approach is superior to two separate laparoscopic approaches. Also, the comparisons with other minimally invasive combinations, such as combined laparoscopic and endo-urologic techniques, should also be studied.[2]
